# Mental health, fatigue and function are associated with increased risk of disease flare following TNF inhibitor tapering in patients with rheumatoid arthritis: an exploratory analysis of data from the Optimizing TNF Tapering in RA (OPTTIRA) trial

**DOI:** 10.1136/rmdopen-2018-000676

**Published:** 2018-05-17

**Authors:** Katie Bechman, Fang En Sin, Fowzia Ibrahim, Sam Norton, Faith Matcham, David Lloyd Scott, Andrew Cope, James Galloway

**Affiliations:** 1 Academic Department of Rheumatology, King’s College London, London, UK; 2 Department of Psychological Medicine, Institute of Psychiatry, Psychology and Neuroscience, KCL, London, UK

**Keywords:** rheumatoid arthritis, anti-tnf, psychology, disease activity

## Abstract

**Background:**

Tapering of anti-tumour necrosis factor (TNF) therapy appears feasible, safe and effective in selected patients with rheumatoid arthritis (RA). Depression is highly prevalent in RA and may impact on flare incidence through various mechanisms. This study aims to investigate if psychological states predict flare in patients’ dose tapering their anti-TNF therapy.

**Methods:**

This study is a post-hoc analysis of the Optimizing TNF Tapering in RA trial, a multicentre, randomised, open-label study investigating anti-TNF tapering in RA patients with sustained low disease activity. Patient-reported outcomes (Health Assessment Questionnaire, EuroQol 5-dimension scale, Functional Assessment of Chronic Illness Therapy fatigue scale (FACIT-F), 36-Item Short Form Survey (SF-36)) were collected at baseline. The primary outcome was flare, defined as an increase in 28-joint count Disease Activity Score (DAS28) ≥0.6 and ≥1 swollen joint. Discrete-time survival models were used to identify patient-reported outcomes that predict flare.

**Results:**

Ninety-seven patients were randomised to taper their anti-TNF dose by either 33% or 66%. Forty-one patients flared. Higher baseline DAS28 score was associated with flare (adjusted HR 1.96 (95% CI 1.18 to 3.24), p=0.01). Disability (SF-36 physical component score), fatigue (FACIT-F) and mental health (SF-36 mental health subscale (MH)) predicted flare in unadjusted models. In multivariate analyses, only SF-36 MH remained a statistically significant predictor of flare (adjusted HR per 10 units 0.74 (95% CI 0.60 to 0.93), p=0.01).

**Conclusions:**

Baseline DAS28 and mental health status are independently associated with flare in patients who taper their anti-TNF therapy. Fatigue and function also associate with flare but the effect disappears when adjusting for confounders. Given these findings, mental health and functional status should be considered in anti-TNF tapering decisions in order to optimise the likelihood of success.

**Trial registration numbers:**

EudraCT Number: 2010-020738-24; ISRCTN: 28955701; Post-results.

**Key messages**

**What is already known about this subject?**

- Anti-tumour necrosis factor (TNF) dose tapering is increasingly used in rheumatoid arthritis, offering significant cost savings.

**What does this study add?**

- One-third of patients who taper their anti-TNF dose will flare over a 12-month period.
- Higher 28-joint count Disease Activity Score score at baseline, along with disability, fatigue and mental health scores, is predictive of flare.

**How might this impact on clinical practice?**

- This information will help physicians make more personalised treatment decisions.

## Background

Disease activity-guided dose tapering or discontinuation of anti-tumour necrosis factor (TNF) therapy appears to be feasible, safe and effective in a selected proportion of patients with rheumatoid arthritis (RA).^1^ However beyond the demonstration of clinical remission by 28-joint count Disease Activity Score (DAS28), there are no standardised methods to identify patients in whom treatment tapering is likely to be successful.^2^ Approximately one to two thirds of patients flare when tapering or stopping anti-TNF treatment.^3^ There is growing evidence that even short-term flare episodes contribute to worsening joint damage^4^ and poorer functional outcome.^5^ The ability to accurately predict who may flare is likely to constitute a major improvement over the current trial-and-error tapering. At present, there are no consistently identified predictive markers for successful dose reduction.^6^

Mental health constitutes a plausible marker for disease flare since poor mental health may influence symptom reporting and interfere with self-management behaviours. Mental health disorder is common in RA with major depression present in 17% of patients and clinically significant depressive symptoms are found in up to 50%.^7^ Worse mental health is associated with increased pain and fatigue^8^ and higher disease activity due to its influence on the tender joint count and the patient global assessment components of the DAS28 score.^9^

Worse mental health is negatively associated with remission in patients on anti-TNF therapy.^10 11^ For those with stable disease, mental health has been identified as an independent factor for flare.^12^ In addition to mental health, concurrent fibromyalgia^13^ and poorer physical quality-of-life measures^14^ have been shown to be associated with an increased risk of flare in patients with low disease activity (LDA) on stable anti-TNF therapy, although it is less clear whether these measures are a reflection of worse mental health and inflammation.

To date, there are no studies directly addressing the role of mental health (depression, anxiety or low mood), fatigue and functional states in predicting flares in patients tapering their biological therapy. The aim of our study was to assess if baseline mental health and functional states measured by self-report screening questionnaires predict flare in RA patients with LDA who undergo treatment tapering of their anti-TNF agent as part of the Optimizing TNF Tapering in RA (OPTTIRA) trial.

## Methods

### Study design and patients

This study is a post-hoc analysis of the OPTTIRA trial. OPTTIRA was a multicentre, prospective, randomised, open-label study investigating anti-TNF tapering in established patients with RA who are in sustained LDA.^15^ The OPTTIRA trial consists of two phases: the randomised, controlled, open-label, proof-of-principle phase (0–6 months), followed by the open exploratory phase (6–12 months) for patients who completed the initial trial period. All patients were receiving anti-TNF agents. They had met existing National Institute for Health and Care Excellence criteria for starting these agents and had achieved a sustained good response, defined as DAS28 scores of ≤3.2 without an increase of >0.6, during the previous  3-month period. Patients were taking either etanercept or adalimumab at standard doses (50 mg/week and 40 mg/fortnight, respectively) and at least one concomitant conventional synthetic disease-modifying antirheumatic drug (DMARD).

**Figure 1:**
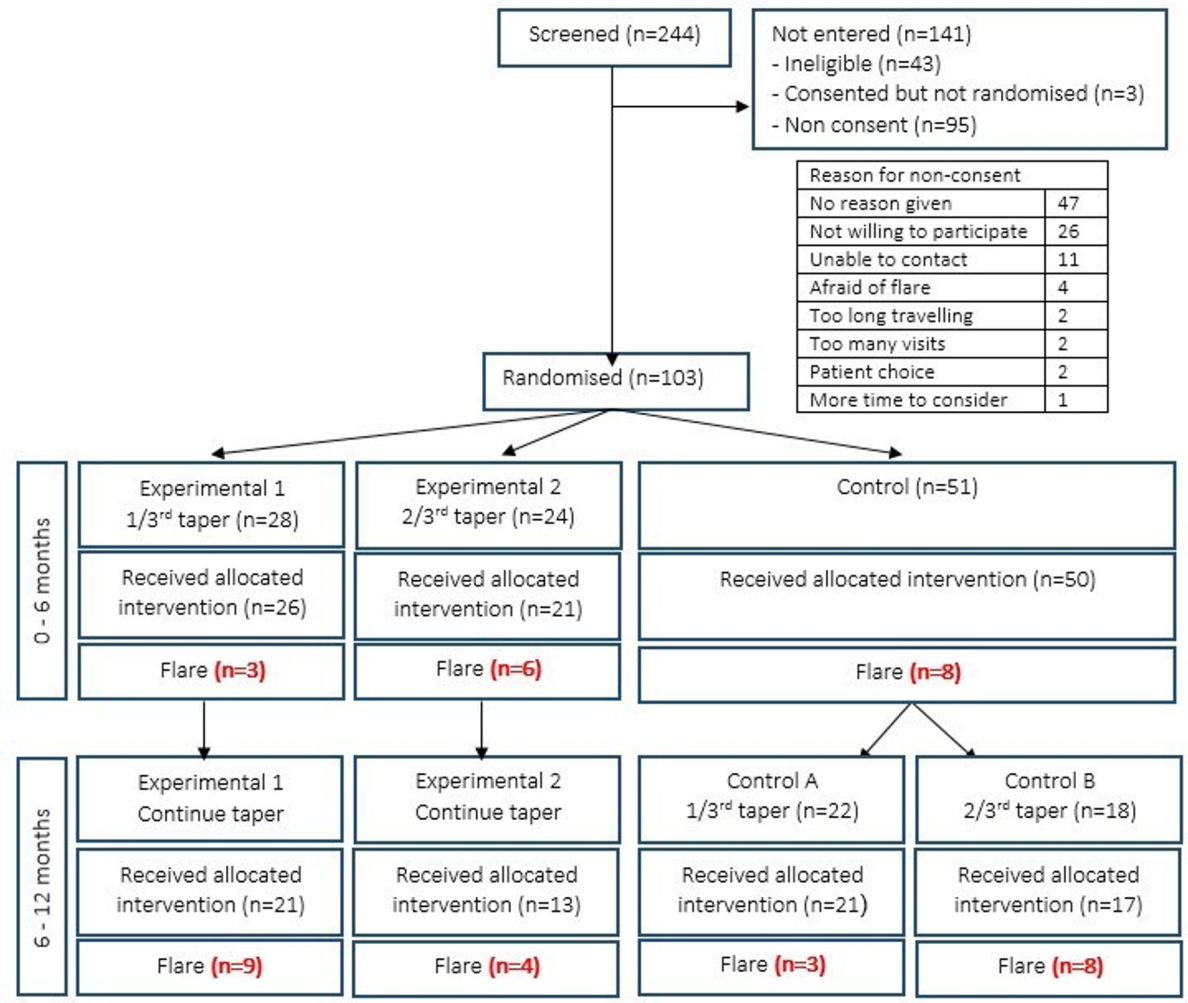
Consort flow chart for Optimizing TNF Tapering in RA trial. IL, interleukin; TNF, tumour necrosis factor.

In the proof-of-principle phase of the study (0–6 months), 50 patients were randomised to a control group (constant anti-TNF dose) and 47 patients to one of two experimental groups—*group 1* (26 patients) tapered anti-TNF by 33% while *group 2* (21 patients) tapered anti-TNF by 66%. At 6 months, patients who had not flared during the initial phase of the study entered a second phase (6—12 months). Those in experimental groups 1 and 2 continued tapering anti-TNF therapy to complete cessation, while patients from the control group were randomised into *control group A* (21 patients) who tapered anti-TNF by 33% or *control group B* (17 patients) who tapered by 66% (figure 1). This post-hoc analysis includes the entire cohort.

Written informed consent was obtained from all patients.

### Clinical assessments

Baseline variables including patient demographics, RA disease duration and concomitant conventional synthetic DMARDs were collected prior to anti-TNF tapering. Disease-related and patient-reported outcome measures that were assessed included the Health Assessment Questionnaire (HAQ), Functional Assessment of Chronic Illness Therapy fatigue scale (FACIT-F), EuroQol 5-dimension scale (EQ-5D-3L) and the 36-Item Short Form Survey (SF-36). Mental health was operationalised using the depression and anxiety question within the EQ-5D and the Mental Health (MH) subscale within the SF-36. The MH subscale shares similarities with generic depression screening questionnaires, such as the Nine-item Patient Health Questionnaire and has been demonstrated to perform reasonably well as a screening tool for depression in RA.^16 17^ A MH score cut-off of ≤56 was validated to detect depression in RA, with a sensitivity and specificity of 92.6% and 73.2%, respectively.^17^

The primary outcome was flare, defined as an increase in DAS28 scores≥0.6 resulting in a DAS28 >3.2. The increase in DAS28 scores must include an increase in swollen joint count and be present on two occasions at least 1 week apart. An increase in DAS28 score ≥1.2 resulting in DAS28 >3.2 between scheduled visits was also defined as flare irrespective of changes in the swollen joint count.

### Statistical analysis

Descriptive statistics were provided with mean (±SD, median (IQR)) or frequencies depending on data distribution.

Discrete-time survival regression models (complementary log–log link) were used to identify predictors of time to flare.^18^ Flare in the previous 3-month interval was the outcome variable and clinical and functional measurements the predictor variables. A multivariate discrete-time survival model was applied adjusting for potential confounders: baseline age, gender, treatment arm, DAS28 and body mass index (BMI). A p value <0.05 was regarded as being statistically significant. As this was an exploratory study, no correction for multiple hypothesis testing was performed. A sensitivity analysis was also performed looking at the predictor variables at the assessment point immediately prior to the flare event.

Missing data were addressed using a multiple imputation module (online online supplementary annex 1). For subjects with missing outcomes, the baseline outcomes and other explanatory covariates (treatment group, sex, age, ethnicity and disease duration) were used to impute the missing data, assuming unobserved measurements were missing at random. All analyses were performed with STATA V.14.1 statistical software.

10.1136/rmdopen-2018-000676.supp1 Supplementary file 1

## Results

### Patient characteristics

Between April 2011 and June 2013, 244 patients were screened, 103 were randomised and 97 accepted their allocated treatment (figure 1). Baseline characteristics are given in table 1. The majority of patients were on methotrexate in combination with their anti-TNF therapy (n=67, 69%) and the median disease duration was 11 years (IQR 7–17). Seventy-three (75%) fulfilled DAS28 remission criteria (DAS28 <2.6).

**Table 1:** Baseline demographics and clinical characteristics

*Demographic variables*	
Patients	97
Age, years*	57 (11)
Female gender	72 (74%)
Disease duration, years†	11.3 (7.3–16.7)
Smoking status	
Ex-smoker	32 (38%)
Current	12 (14%)
BMI	25.4 (22.6–29.4)
*Clinical variables*	
Treatment csDMARD	
Methotrexate	67 (69%)
Hydroxychloroquine	7 (7%)
Sulfasalazine	4 (4%)
Leflunomide	4 (4%)
csDMARD combination	15 (15%)
Treatment bDMARD	
Adalimumab	54 (56%)
Etanercept	43 (44%)
Radiographic damage (Larsen score)†	51 (16–82)
Tender joint counts (28 joints)†	0 (0–1)
Swollen joint counts (28 joints)†	0 (0–0)
Patient global assessment (mm)†	5 (1–16)
Erythrocyte sedimentation rate(mm/hour)†	8 (5–19)
C-reactive protein (mg/L)†	5 (2–6)
DAS28-ESR†	2.0 ()
DAS28-ESR<2.6 (remission)	73 (75%)
*Mental health variables*	
Health Assessment Questionnaire score†	0.50 (0.13–1.38)
EQ-5D-3L score†	0.76 (0.66–1.00)
EQ-5D-3L depression question	21 (22%)
FACIT fatigue scale	41 (35–46)
SF-36	
Physical component summary*	45 (34 – 52)
Mental component summary*	57 (49 – 60)
SF-36 MH score†	84 (72–92)
SF-36 MH (score<56)	10 (10%)
*Treatment arm*	
Experimental 1 (taper 1/3rd)	26 (27%)
Experimental 2 (taper 2/3rd)	21 (22%)
Control A (taper 1/3rd)	27 (28%)
Control B (taper 2/3rd)	23 (24%)

All values are gives as number (%) unless otherwise specified.

*Mean (SD).

†Median (p25–p75).

BMI, body mass index; bDMARD, biological DMARD; csDMARD, conventional synthetic DMARD; EQ-5D, EuroQol 5-dimension scale; FACIT-F, Functional Assessment of Chronic Illness Therapy fatigue scale; SF-36 MH, SF-36 Mental Health subscale.

HAQ scores >1, suggesting moderate to severe disability, were observed in 34% of patients. Median HAQ score was 0.5 (IQR 0.13–1.38). The median EQ-5D score was 0.76 (0.66–1.00) on a scale, where higher scores represent better quality of life. Twenty-two per cent of the cohort admitted to feeling symptoms of depression and anxiety on the EQ-5D depression question. Patients scored higher on the mental component of the SF-36 than the physical component (57 (49–60) vs 45 (34–52)), on a scale of 0–100 where higher scores represent better health states. The median SF-36 MH subscale score was 84. A score of ≤56, the MH cut-off used to detect depression was observed in 11% of patients.

### Characteristics of flare

Forty-one patients (42%) flared over the 12-month period. In the first phase of the study (0–6 months), three patients who tapered anti-TNF by 33% (experimental group 1) and six patients who tapered anti-TNF by 66% (experimental group 2) flared, while eight patients in the control arm flared (figure 1).

In the second phase of the study (6–12 months), nine patients in experimental group 1 and four patients in experimental group 2 who were continuing tapering of anti-TNF therapy to complete cessation flared. In the control group, three patients who tapered anti-TNF by 33% (control group A) and eight patients who tapered anti-TNF by 66% (control group B) flared.

There was a statistically significant difference in baseline SF-36 MH and FACIT-F scores between patients who flared compared with those who did not; MH score (flare: 80 (68–88) vs no flare: 88 (72–92), p=0.04) and FACIT-F score (flare: 39 (31–44) vs no flare: 43 (37–46), p=0.03). The lower the score the more severe the symptoms of depression or fatigue, respectively. There was a greater proportion of patients categorised with depression in the flare group by MH score ≤56 (8% vs 3%, p=0.03), but there was no difference when depression was categorised by the EQ-5D anxiety and depression question (11% vs 10%, p=0.29). There were no differences in baseline EQ-5D or HAQ score between the two groups.

### Prediction of flare

Our primary analyses considered baseline patient characteristics in a flare prediction model (table 2). A higher DAS28 score at study entry was associated with increased hazard for flare. This association remained significant even after adjusting for co-variates (HR 1.96 (95% CI 1.18 to 3.24), p=0.04).

**Table 2:** Unadjusted and adjusted HRs for flare

	Unadjusted HRs	
	HR (95% CI)	P values
*Demographic variables*		
Age, years	1.02 (0.99 to 1.04)	0.24
Gender (male)	0.87 (0.42 to 1.82)	0.72
Disease duration, years	1.01 (0.97 to 1.04)	0.78
BMI	1.03 (0.97 to 1.09)	0.38
Treatment arm		
Taper 1/3rd	1.28 (0.55 to 2.98)	0.57
Taper 2/3rd	2.51 (1.06 to 5.96)	0.04
*Clinical variables*		
DAS28		
Unadjusted	1.86 (1.19 to 2.92)	0.01
Adjusted (age, gender, trial arm)	1.96 (1.18 to 3.24)	0.01
*Mental health variables*		
HAQ-DI		
Unadjusted	1.45 (0.99 to 2.13)	0.06
Adjusted (age, gender, trial arm)	1.43 (0.91 to 2.29)	0.13
Adjusted (age, gender, trial arm, BMI, DAS28)	1.16 (0.72 to 1.87)	0.53
EQ-5D		
Unadjusted	0.28 (0.07 to 1.24)	0.09
Adjusted (age, gender, trial arm)	0.29 (0.06 to 1.38)	0.12
Adjusted (age, gender, trial arm, BMI, DAS28)	0.51 (0.10 to 2.58)	0.42
EQ-5D depression anxiety		
Unadjusted	1.42 (0.70 to 2.87)	0.33
Adjusted (age, gender, trial arm)	1.37 (0.64 to 2.96)	0.41
Adjusted (age, gender, trial arm, BMI, DAS28)	1.51 (0.70 to 3.28)	0.29
FACIT-F (per 10 unit)		
Unadjusted	0.68 (0.47 to 0.99)	0.04
Adjusted (age, gender, trial arm)	0.78 (0.48 to 1.14)	0.18
Adjusted (age, gender, trial arm, BMI, DAS28)	0.77 (0.50 to 1.16)	0.20
SF-36 PCS (per 10 unit)		
Unadjusted	0.74 (0.55 to 0.99)	0.05
Adjusted (age, gender, trial arm)	0.72 (0.52 to 1.00)	0.05
Adjusted (age, gender, trial arm, BMI, DAS28)	0.86 (0.60 to 1.23)	0.41
SF-36 MCS (per 10 unit)		
Unadjusted	0.90 (0.62 to 1.31)	0.58
Adjusted (age, gender, trial arm)	0.93 (0.60 to 1.44)	0.74
Adjusted (age, gender, trial arm, BMI, DAS28)	0.83 (0.54 to 1.28)	0.41
SF-36 MH (per 10 unit)		
Unadjusted	0.81 (0.67 to 0.96)	0.01
Adjusted (age, gender, trial arm)	0.80 (0.65 to 0.98)	0.03
Adjusted (age, gender, trial arm, BMI, DAS28)	0.75 (0.60 to 0.93)	0.01

BMI, body mass index; DAS28, 28-joint count Disease Activity Score; EQ-5D, EuroQol 5-dimension scale; FACIT, Functional Assessment of Chronic Illness Therapy fatigue scale; HAQ-DI, Health Assessment Questionnaire Disability Index; SF36 MCS, mental component summary; SF36 MH, Mental Health subscale; SF36 PCS, physical component summary.

Disability (SF-36 physical component) predicted flare in the unadjusted model (HR per 10 units 0.74 (95% CI 0.55 to 0.99), p=0.05). Fatigue (FACIT-F) and mental health (SF-36 MH) also predicted flare in univariate models (FACIT-F HR per 10 units 0.68 (95% CI 0.47 to 0.99), p=0.04) (MH HR per 10 units 0.81 (95% CI 0.68 to 0.96), p=0.01). In adjusted analyses, only MH remained a statistically significant predictor of flare (HR per 10 units 0.74 (95% CI 0.60 to 0.93), p=0.01). HAQ was not a statistically significant predictor of flare, although the direction of association was consistent.

We also analysed the predictor variables at the assessment point immediately prior to the flare event. We use this time-dependant analysis to determine whether flare is predicted by variables measures at closer time points to the event. There was no clinically meaningful difference in the point estimates of effects (online supplementary data). The imputation model confirmed these findings.

## Discussion

To our knowledge, this is the first study to date to investigate the effect of mental health and functional states on the risk of flare when tapering anti-TNF therapy in patients with RA. Disability, fatigue and mental health as measured by patient-reported outcomes including SF-36 physical component, FACIT-F and the SF-36 mental health subscale predicted flare. Mental health as defined by the SF-36 MH was the only independent predictor of flare after adjusting for age, gender, treatment arm, DAS28 and BMI. The MH score ranges from 0 to 100, with lower scores indicating more severe depressive symptoms. With every 10-point decrease in MH score, the risk of flare increases by 19%. Both HAQ and EQ-5D were not statistically significant predictors of flare, although the direction of association was consistent.

Unlike the other variables, the SF-36 MH subscale specifically assesses depressive symptoms with items relating to low mood, nerves and restlessness. It shares similarities with generic depression screening tools, such as the PHQ9.^17^ In comparison, the other baseline measures assess quality of life and general mental health. For example, the SF-36 mental component summary is calculated by positively weighting the MH and three other psychological subscales (vitality, social function, emotional role). This suggests that depression alone can independently predict flare in patients who taper their anti-TNF agents. Depression can impact patients’ perception and interpretation of their symptoms^19^ and is associated with poor health behaviour including reduced treatment adherence.^20^ There is limited literature on the impact of depression in RA tapering cohorts. In patients who remain on stable treatment, depression has been shown to predict future disease activity, flare^12 21^ and a poorer response to treatment.^22 23^ In drug tapering studies, HAQ is the only patient-reported measure that has been evaluated, with lower scores associated with successful tapering in univariate analyses.^24–26^

The nocebo effect is a well-known phenomenon where patients’ concerns and expectations about the value of a therapeutic intervention negatively influence adherence and treatment response. This has been considered in patients switching biologics from bio-originators to biosimilars, to explain a deterioration in therapeutic benefit,^27^ although the clinical features are complex and undefined.^28^ It is acknowledged that patients with mental illness are more susceptible to the nocebo effect^27^ and it is plausible that this may also contribute to the association between unsuccessful drug tapering and flare in patients with poor mental health.

In this study, univariate analyses demonstrated that measures of quality-of-life status helped predict flare. However in the adjusted model, these measures did not remain statistically significant predictors. It is possible that measures of psychological and functional well-being correlate with other factors in a causal pathway; for example, fatigue affects components of the DAS28 score, increasing the overall score and amplifying the risk of flare. Thus when adjusted for DAS28, the predictive value of these measures is lost. The direction of effect does not change in the adjusted model and it is likely that the loss in statistical significance is related to a loss of power due to the limited OPTTIRA sample size.

A higher DAS28 score at entry was also predictive of flare in this study. The current literature on the predictive value of DAS28 at point of anti-TNF tapering is conflicting. DAS28 was found to be a predictor of successful drug tapering in only half of the studies in which it was evaluated.^6^ In two anti-TNF discontinuation studies (remission induction by Remicade in RA^29^ and the HONOR study^30^), analyses indicated a lower DAS28 cut-off value of 2.22 and 1.98, respectively, was required for successful drug tapering. The OPTIRA patient cohort was an LDA cohort, in which a quarter of patients’ baseline DAS28 scores were greater than the remission cut-off of 2.6. This may explain why DAS28 was shown to be a strong predictor of flare compared with exclusive remission cohorts.

When considering these findings, it is important to note the limited success in identifying biomarkers that predict dose tapering. Serological status (anti-cyclic citrullinated peptide antibodies),^31^ ultrasound Doppler-detected synovitis^31 32^ and the multi-biomarker disease activity score^33^ have been individually evaluated. Although positive findings should be interpreted with caution due to reporting bias and multiple testing. A systematic review of all tapering studies identified adalimumab through level, the Sharp/van der Heijde erosion score and duration of symptoms at start of biologic to predict successful tapering.^6^

The proportion of patients with depression in this population, defined by MH score ≤56, was relatively low. Clinical remission may be a significant influence of improvement in mental health states for patients both with and without baseline depression. It is recognised that patients who achieve clinical remission experience improvements in their depression and anxiety symptoms.^21^ This may be due to reduction in pain and fatigue levels from control of RA disease activity or it may be directly attributable to a reduction in pro-inflammatory cytokines including TNF-α, which can modulate neurotransmitter systems.^34^ It is possible that consent bias resulted in the inclusion of ‘happier’ patients, who are less likely to suffer from mental health disorder. Of the 244 patients screened, only 103 consented and entered randomisation (consort flow chart). It has been reported that consenters are less likely to have a sensitive diagnosis such as a mood disorder,^35 36^ and those who do are less likely to continue participation in clinical studies and can contribute to missing data.

Lastly, the OPTTIRA trial used a stringent definition of flare, which included the requirement for at least one swollen joint count to account for the increase in DAS28. In prior studies identifying an association between psychological measures and disease activity, the increase in DAS28 score has been driven primarily by tender joint count or global assessment score^22^ which may be influenced by psychosocial factors.^37^ In contrast, the captured flare events in the OPTTIRA study are more likely to represent a genuine inflammatory disease flare and less likely influenced by low mood or depression. The OMERACT RA flare group recognises the limitation of DAS28 in defining flare events. There is disparity between the classification of a flare by a patient, their physician and the DAS28 criteria. Agreement across these classifications is higher in patients in remission or LDA.^38^ A consensus-based core domain has been developed to provide a greater patient-centred tool to identify and measure flare in RA.^38 39^ Improving the definition of flare may help identify and precisely quantify inflammatory flares which is vital in guiding successful drug tapering.

This study has several strengths. OPTTIRA was a pragmatically designed study, with less stringent inclusion and exclusion criteria and thus the cohort is far more representative than a highly selective clinical trial population. The inclusion of patients with LDA in addition to those in remission increases the generalisability of our findings. Lastly, this was a deeply phenotyped cohort with extensive clinical and laboratory data at multiple time points across the study period including precise date of flare events.

There are potential limitations to this study. First, we must acknowledge the limitation of the OPTTIRA study sample size. The failure to detect other predictors of flare could reflect a type II error and the study’s lack of power preclude robust conclusions. The high scores from the MH compared with population point-prevalence estimates may reflect that our sample size was not large enough to capture sufficient patients with depression. Second, the study duration was relatively short and may not have provided a long enough period to allow patients to flare. We did not record or analyse sustained flares which may prove more important than potential transient flares. Lastly, there are a multitude of methods available to detect health-related quality of life and depression. The gold standard method for diagnosis of depression is psychiatric interview and diagnosis according to Diagnostic and Statistical Manual or International Classification of Diseases criteria. Despite using both a disease-specific assessment (HAQ) and generic measures applicable to both the normal population and other disease groups (SF-36 and EQ-5D), these are ultimately only screening tools. Estimates according to screening tools are based on predefined thresholds and tend to prioritise sensitivity over specificity which may result in overestimations of prevalence of depression.^7^

## Conclusion

Baseline depression, measured by SF-36 mental health scale and DAS28, independently predict flare events in patients with sustained LDA who taper their anti-TNF agents. In addition to baseline depression, a range of psychological and functional states measured by patient-reported outcomes also predicted flare events in the OPTIRRA cohort although these were not be demonstrated to be independent risk factors. Based on these findings, an assessment of mental health and functional status should be considered prior to dose reduction.
