# Supplementary material for: Mental health, fatigue and function are associated with increased risk of disease flare following TNF inhibitor tapering in patients with rheumatoid arthritis: an exploratory analysis of data from the Optimizing TNF Tapering in RA (OPTTIRA) trial
Source: RMD Open. 2018 May 17;4(1):e000676. doi: 10.1136/rmdopen-2018-000676 (PMC5976130; doi:10.1136/rmdopen-2018-000676)
Supplement: Supplementary file 1 [file rmdopen-2018-000676supp001.docx]

**Annex 1:** Imputation methodology

There was few baseline data missing [Larsen X-ray score n=1]. At 3 and 6, month time points, there were less than 4% missing data, which were mainly psychological and functional measures, and around 10% missing data at 12 months. At 9 month, no psychological or functional measures were captured and there was 23% data missing on clinical variables**.** These data was considered missing at random.

| Variable | Observations | Missing Variables |
| --- | --- | --- |
| Baseline X-ray score | 96 | 1 |
| Tender joint count | 448 | 37 |
| Swollen joint count | 449 | 36 |
| ESR | 447 | 38 |
| VAS | 446 | 39 |
| DAS-28 | 447 | 38 |
| CRP | 449 | 36 |
| Patient global assessment | 448 | 37 |
| PAIN score | 446 | 39 |
| HAQ-DI | 371 | 114 |
| EQ5D score | 376 | 109 |
| EQ5D Depression and anxiety question | 435 | 50 |
| FACIT-F | 372 | 113 |
| SF36-PCS | 372 | 113 |
| SF36-MCS | 372 | 113 |
| MH score | 357 | 128 |

The disease activity score for 28 joints and its component were imputed using multivariate sequential imputation using chained equations. Firstly, all missing values were filled in by simple random sampling with replacement from the observed values. The first variable with missing values, say tender joint count at visit one, was regressed on all other variables. Missing values in tender joint count-1 were replaced by simulated data points drawn from the corresponding posterior predictive distribution of tender joint count-1. Then, the next variable with missing was replaced by the same cycle. The imputation was 20 cycles, where at the end of the cycle one imputed dataset was created and the process was repeated to create 20 imputed datasets. The 20 datasets were combined using Rubin’s rules [1, 2] therefore, the estimates and standard errors presented here are the combined ones.

References

1. Little RJA, Rubin DB. Statistical Analysis with Missing Data. 2nd ed. Hoboken, NJ: John Wiley and Sons, Inc; 2002

2. Schafer JL. Analysis of Incomplete Multivariate Data. 1st ed. London, United Kingdom: Chapman and Hall Ltd; 1997.

**Annex 2:** Full analysis with unadjusted and adjusted hazard ratios (HR) for flare

|  | Cox regression | | Discrete time survival regression models (complementary log-log link) | | | | Imputation  Discrete time survival regression models (complementary log-log) | |
| --- | --- | --- | --- | --- | --- | --- | --- | --- |
|  |  |  | Baseline variables | | Last observation prior to flare | |  |  |
|  | **HR (95% CI)** | ***p*** | **HR (95% CI)** | ***p*** |  | | **HR (95% CI)** | ***p*** |
| Age, years | 1.01 (0.99-1.04) | 0.35 | 1.02 (0.99-1.04) | 0.24 |  |  | 1.02 (0.99-1.05) | 0.24 |
| Gender (male) | 0.74 (0.35-1.56) | 0.43 | 0.87 (0.42-1.82) | 0.72 |  |  | 0.88 (0.43-1.82) | 0.73 |
| Disease duration, years | 1.01 (0.97-1.04) | 0.73 | 1.01 (0.97-1.04) | 0.78 |  |  | 1.00 (0.97-1.04) | 0.78 |
| BMI | 1.02 (0.97-1.09) | 0.37 | 1.03 (0.97-1.09) | 0.38 |  |  | 1.03 (0.97-1.09) | 0.38 |
| Treatment arm  - Taper 1/3^rd^  - Taper 2/3^rd^ | -  1.65 (0.85-3.21) | -  0.14 | 1.28 (0.55-2.98)  **2.51 (1.06-5.96**) | 0.57  **0.04** |  |  | 1.29 (0.55-2.98)  **2.51 (1.06-5.96)** | 0.57  **0.04** |
| ***Clinical variables*** | | | | | | | | |
| DAS28  - unadjusted  - adjusted (age, gender, trial arm) | **1.84 (1.19, 2.84)**  **1.86 (1.19, 2.91)** | **0.01**  **0.01** | **1.86 (1.19, 2.92)**  **1.96 (1.18, 3.24)** | **0.01**  **0.01** | **2.01 (1.45-2.78)**  **2.09 (1.51, 2.89)** | **0.00**  **0.00** | **2.02 (1.45-2.80)**  **2.11 (1.53, 2.92)** | **0.00**  **0.00** |
| ***Mental health variables*** | | | | | | | | |
| HAQ-DI  - unadjusted  - adjusted (age, gender, trial arm)  - adjusted (age, gender, trial arm, bmi, das28) | 1.38 (0.94-2.04)  1.31 (0.85, 2.01)  1.01 (0.62, 1.64) | 0.10  0.22  0.96 | 1.45 (0.99, 2.13)  1.43 (0.91, 2.29)  1.16 (0.72, 1.87) | 0.06  0.13  0.53 | **1.52 (1.01-2.29)**  1.46 (0.90-2.38)  1.14 (0.69-1.90) | **0.04**  0.12  0.61 | **1.51 (1.04-2.18)**  1.48 (0.94-2.33)  1.13 (0.71-1.80) | **0.03**  0.09  0.61 |
| EQ-5D  - unadjusted  - adjusted (age, gender, trial arm)  - adjusted (age, gender, trial arm, bmi, das28) | 0.42 (0.08, 2.15)  0.46 (0.08, 2.48)  0.86 (0.15, 5.11) | 0.30  0.36  0.87 | 0.28 (0.07, 1.24)  0.29 (0.06, 1.38)  0.51 (0.10, 2.58) | 0.09  0.12  0.42 | **0.22 (0.05-0.96)**  0.27 (0.05-1.36)  0.53 (0.09-3.03) | **0.04**  0.11  0.48 | **0.21 (0.05-0.87)**  0.23 (0.05-1.13)  0.50 (0.10-2.55) | **0.03**  0.07  0.40 |
| EQ-5D depression anxiety  - unadjusted  - adjusted (age, gender, trial arm)  - adjusted (age, gender, trial arm, bmi, das28) | 1.35 (0.73, 2.52)  1.16 (0.61, 2.20)  1.20 (0.62, 2.28) | 0.34  0.64  0.59 | 1.42 (0.70, 2.87)  1.37 (0.64, 2.96)  1.51 (0.70, 3.28) | 0.33  0.41  0.29 | 1.74 (0.81-3.78)  1.66 (0.75-3.68)  1.63 (0.75-3.55) | 0.16  0.21  0.22 | 1.96 (0.91-4.21)  1.85 (0.83-4.14)  1.76 (0.82-3.77) | 0.09  0.13  0.15 |
| FACIT: (per 10 unit)   - unadjusted - adjusted (age, gender, trial arm) - adjusted (age, gender, trial arm, bmi, das28 | **0.71 (0.50-1.00)**  0.79 (0.55-1.13)  0.86 (0.60-1.28) | **0.05**  0.19  0.49 | **0.68 (0.47, 0.99)**  0.78 (0.48, 1.14)  0.77 (0.50, 1.16) | **0.04**  0.18  0.20 | 0.71 (0.49-1.03)  0.74 (0.50-1.11)  0.82 (0.56-1.19) | 0.07  0.15  0.30 | 0.72 (0.51-1.03)  0.76 (0.52-1.10)  0.83 (0.58-1.18) | 0.07  0.15  0.29 |
| SF-36 PCS: (per 10 unit)   - unadjusted - adjusted (age, gender, trial arm) - adjusted (age, gender, trial arm, bmi, das28 | 0.78 (0.58-1.06)  0.75 (0.55-1.03)  0.92 (0.64-1.32) | 0.11  0.08  0.63 | **0.97 (0.94-1.00)**  **0.97 (0.94-1.00)**  0.99 (0.95-1.02) | **0.05**  **0.05**  0.41 | **0.73 (0.53-1.00)**  0.73 (0.52-1.05)  0.93 (0.60-1.42) | **0.05**  0.09  0.73 | 0.78 (0.58-1.07)  0.79 (0.56-1.11)  1.02 (0.69-1.51) | 0.13  0.17  0.93 |
| SF-36 MCS: (per 10 unit)   - unadjusted - adjusted (age, gender, trial arm) - adjusted (age, gender, trial arm, bmi, das28) | 0.93 (0.65-1.31)  0.96 (0.67-1.39)  0.88 (0.61-1.27) | 0.66  0.85  0.50 | 0.90 (0.62, 1.31)  0.93 (0.60, 1.44)  0.83 (0.54, 1.28) | 0.58  0.74  0.41 | **0.75 (0.56-1.00)**  0.75 (0.55-1.03)  0.77 (0.57-1.04) | **0.05**  0.08  0.09 | 0.74 (0.54-1.01)  0.75 (0.54-1.05)  0.75 (0.55-1.04) | 0.06  0.09  0.08 |
| SF-36 MH: (per 10 unit)   - unadjusted - adjusted (age, gender, trial arm) - adjusted (age, gender, trial arm, bmi, das28) | **0.83 (0.69-0.99)**  0.84 (0.69-1.02)  **0.80 (0.66-0.98)** | **0.04**  0.07  **0.03** | **0.81 (0.67, 0.96)**  **0.80 (0.65, 0.98)**  **0.75 (0.60, 0.93**) | **0.01**  **0.03**  **0.01** | 0.84 (0.69-1.03)  0.85 (0.68-1.05)  0.85 (0.68-1.05) | 0.09  0.13  0.13 | **0.82 (0.68-0.99)**  0.83 (0.67-1.01)  0.83 (0.67-1.02) | **0.04**  0.07  0.08 |

Abbreviations: BMI, body mass index; DAS28, 28-joint count Disease Activity Score; EQ-5D, EuroQol 5-dimension scale; FACIT-F, Functional Assessment of Chronic Illness Therapy fatigue scale; HAQ-DI, Health Assessment Questionnaire Disability Index, SF36 PCS; 36-Item Short Form Survey Physical Component Summary, SF36 MCS; 36-Item Short Form Survey Mental Component Summary; SF36 MH; Mental Health subscale

**Annex 3:** Figure 2: Forest plot of unadjusted hazard ratios for flare. (Graphical representation of results from table 2)


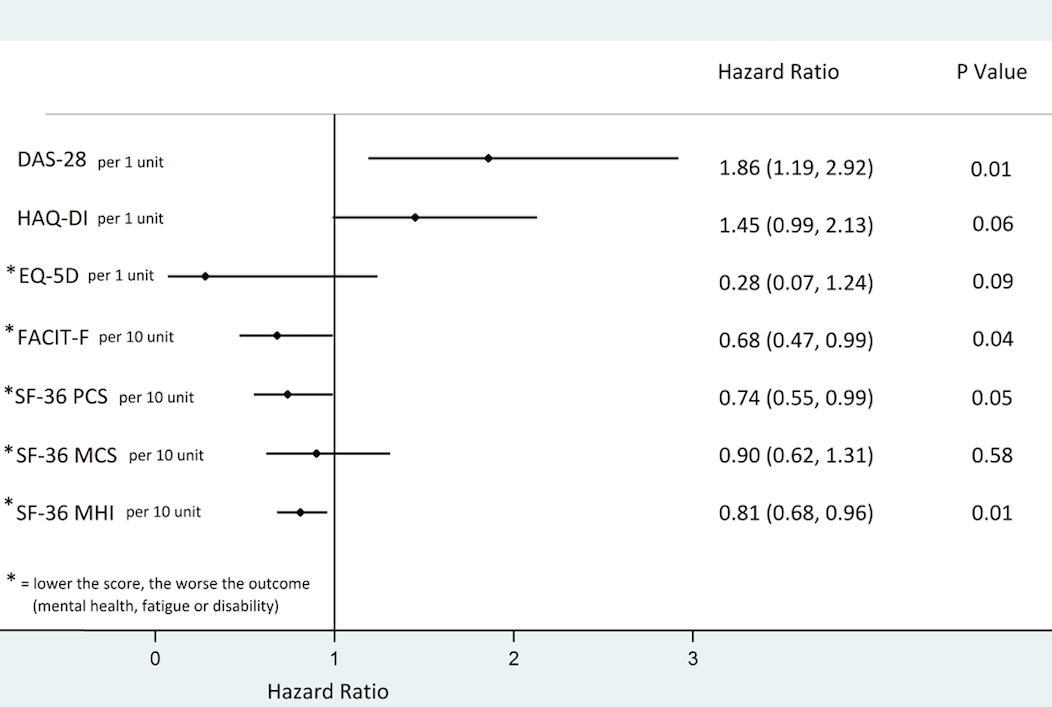


Abbreviations: DAS28, 28-joint count Disease Activity Score; EQ-5D, EuroQol 5-dimension scale; FACIT-F, Functional Assessment of Chronic Illness Therapy fatigue scale; HAQ-DI, Health Assessment Questionnaire Disability Index, SF36 PCS; 36-Item Short Form Survey Physical Component Summary, SF36 MCS; 36-Item Short Form Survey Mental Component Summary; SF36 MH; Mental Health subscale
